# Psychedelics for depression: from neurobiology to treatment

**DOI:** 10.1192/j.eurpsy.2023.74

**Published:** 2023-07-19

**Authors:** K. P. Kuypers

**Affiliations:** Neuropsychology and Psychopharmacology, Maastricht University, Maastricht, Netherlands

## Abstract

**Abstract:**

Decades ago, the classical psychedelics psilocybin and LSD entered the therapeutic setting and already then showed their therapeutic potential in the treatment of psychiatric disorders. For thousands of years another psychedelic, ayahuasca, is being used by tribes in western Amazonia for healing and divination, and in recent years its use has expanded worldwide.

Research into the therapeutic potential of these substances has re-emerged and (preliminary) findings are promising, showing that after one or two administrations remission is reached in depressed patients that were labeled as treatment-resistant. This is a remarkable finding as the therapeutic effects of treatment with conventional pharmacological agents like SSRIs take longer to lead to remission, with one-third of the patients failing to reach this stage.

The fast onset of positive therapeutic effects by psychedelics increases the interest to discover the mechanism of action behind this. There is a debate about the importance of the psychological experience caused by these agents in the therapeutic outcome, while science also tries to understand the neurobiological correlates. The latter will be addressed in my talk and I will link it to psychedelics’ therapeutic effects.

**Disclosure of Interest:**

None Declared

